# Exploring the Phytochemistry, Signaling Pathways, and Mechanisms of Action of *Tanacetum parthenium* (L.) Sch.Bip.: A Comprehensive Literature Review

**DOI:** 10.3390/biomedicines12102297

**Published:** 2024-10-10

**Authors:** Ali Kashkooe, Atefeh Jalali, Mohammad M. Zarshenas, Azadeh Hamedi

**Affiliations:** 1 Medicinal Plants Processing Research Center, Shiraz University of Medical Sciences, Shiraz 71468-64685, Iran; ali.kashkooe@yahoo.com (A.K.);; 2 Department of Phytopharmaceuticals (Traditional Pharmacy), School of Pharmacy, Shiraz University of Medical Sciences, Shiraz 71345-1583, Iran; 3 Department of Pharmacognosy, Shiraz University of Medical Sciences, Shiraz 71468-64685, Iran

**Keywords:** feverfew, phytochemical, signaling pathway, *Tanacetum parthenium*

## Abstract

The traditional use of *Tanacetum parthenium* (L.) Sch.Bip., commonly known as feverfew, extends across various medical conditions, notably those associated with pain and inflammation. In alignment with the growing trend towards developing medications that target specific signaling pathways for enhanced efficacy and reduced side effects, extensive research has been conducted to investigate and validate the pharmacological effects of feverfew. Among its bioactive compounds, parthenolide stands out as the most potent, categorized as a germacranolide-type sesquiterpene lactone, and has been extensively studied in multiple investigations. Significantly, the anti-inflammatory properties of feverfew have been primarily attributed to its capacity to inhibit nuclear factor-kappa B (NF-κB), resulting in a reduction in pro-inflammatory cytokines like tumor necrosis factor-alpha (TNF-α). Furthermore, the anticancer properties of feverfew have been associated with the modulation of Mitogen-Activated Protein Kinase (MAPK) and NF-κB signaling pathways. This study further delves into the neuroprotective potential of feverfew, specifically in the management of conditions such as migraine headaches, epilepsy, and neuropathic pain through various mechanisms. The core objective of this study is to elucidate the phytochemical composition of feverfew, with a particular emphasis on understanding the molecular mechanisms and examining the signaling pathways that contribute to its pharmacological and therapeutic effects. Additionally, the safety, toxicity, and potential adverse effects of feverfew are comprehensively evaluated, with an overarching goal of providing valuable insights into the plant’s potential for targeted and effective treatments.

## 1. Introduction

*Tanacetum parthenium* (L.) Sch.Bip., commonly known as feverfew, belongs to one of the largest families of flowering plants, named Asteraceae [1,2]. ‘Feverfew’ originates from the Latin word ‘febrifugia’, meaning ‘fever reducer’ [2]. In terms of its physical characteristics, *T. parthenium* is a small, bushy, aromatic perennial plant typically growing between 0.3 and 1 m tall. It has yellow-green alternate leaves that are usually under 8 cm long, displaying almost hairless, chrysanthemum-like features. Its vivid yellow flowers, around 2 cm in diameter, form a compact cluster with a flat top, blooming typically from July through October [3]. Notably, the plant species originates from the Balkan Peninsula but can be found in various regions worldwide, including Asia, Europe, North America, Australia, and North Africa [2,3]. It has traditionally been prescribed in ethnic and complementary medicine for a variety of medical purposes, including spasm, anxiety, arthritis, asthma, colic, depressive disorders, epilepsy, fever, and inflammatory conditions [2,4].

Resembling chamomile (*Matricaria chamomilla*), feverfew’s flowers emit a strong, bitter scent, which has led to its use under various aliases such as wild chamomile, chamomile grande, and *Matricaria eximia* hort. It is also known by a plethora of other common names, including featherfoil, parthenolide, bachelor’s button, *Chrysanthemum parthenium*, altamisa, febrifuge plant, midsummer daisy, nosebleed, Santa Maria, wild quinine, *Chrysanthemum atricaire*, flirtwort, federfoy, mother herb, *Leucanthemum parthenium*, *Parthenium hysterophorus*, *Pyrenthrum parthenium* L., flirtroot, feddygen fenyw, mutterkraut, and vetter-voo. This wide array of names reflects the diverse nomenclature and versatile applications associated with this plant [3].

*T. parthenium* is composed of a variety of metabolites such as sesquiterpenes, monoterpenes, flavonoid glycosides, and various other compounds [5]. It is especially rich in sesquiterpene lactones, with over 30 distinct types identified in the plant, all characterized by a 15-carbon structure. A notable sesquiterpene lactone in *T. parthenium* is ‘Parthenolide’, which belongs to the germacranolide-type and is prominently present in the leaves, seeds, and flowers. This compound plays a crucial role in the plant’s pharmacological activities due to its high concentrations [6].

Identifying the common target pathways of a therapeutic agent is considered the first step in developing targeted and efficient drugs [7]. Therefore, various studies have been conducted on medicinal plants, especially those traditionally prescribed, to discover underlying mechanisms of action and signaling pathways. As feverfew is well known for its anti-inflammatory effects, signaling pathways related to inflammation, such as nuclear factor-kappa B (NF-κB), have undergone the most extensive study. This transcription factor plays a crucial role in various biological pathways, such as inflammation and immune response, as well as in cell proliferation, survival, and apoptosis [8]. Another important therapeutic effect of feverfew would be its anticancer activity. The mitogen-activated protein kinase (MAPK) pathway also plays a crucial role in cell proliferation, differentiation, and stress responses [9].

This review article explores feverfew’s phytochemical components, signaling pathways, and mechanisms of action for its pharmacological effects. Additionally, the aim was to provide crucial insights from the current literature on targeted treatments, with a focus on molecular mechanisms, as well as an evaluation of the safety, toxicity, and potential adverse effects of feverfew.

## 2. Literature Review Methodology

In conducting a meticulous search strategy, the keywords “*Tanacetum parthenium*” and “feverfew” were thoroughly explored up to the timeframe of August 2024 across well-established databases, including Medline, PubMed, and Scopus, leading to the initial discovery of 993 relevant papers on *T. parthenium*. Through implementing stringent selection criteria, studies related to genetic and agricultural sciences, environmental sciences, nursing, and content categorized as reviews, letters, or conference materials were omitted from this literature review, with a specific emphasis on papers written in the English language. Subsequently, two independent reviewers carefully scrutinized all chosen articles, extracting crucial information concerning the metabolites present in various parts of the feverfew plant, its potential medicinal properties, mechanisms of action or signaling pathways, and comprehensive details on its safety, toxicity, and potential adverse effects. By conducting a meticulous data analysis, this narrative review aimed to offer an in-depth exploration of the key findings related to feverfew in the scientific literature.

## 3. Phytochemistry

A variety of metabolites have been identified from different parts of the feverfew plant. The most extensively studied classes of phytochemicals found in feverfew include sesquiterpene lactones and flavonoids. Additionally, other compounds present in the plant belong to phytosterols, triterpenoids, fatty acids, phenolic acids, and essential oils.

### 3.1. Sesquiterpene Lactones

Sesquiterpene lactones are a class of sesquiterpenoids that contain a lactone ring. Sesquiterpene lactones are the most important secondary metabolites of feverfew. Based on the chemical ring structures, these compounds are classified mainly into eudesmanolides, germacranolides, and guaianolides. Parthenolide, a germacranolide, is the major sesquiterepene lactone of this plant, composed of up to 85% of the total sesquiterpene.

**Figure 1:**
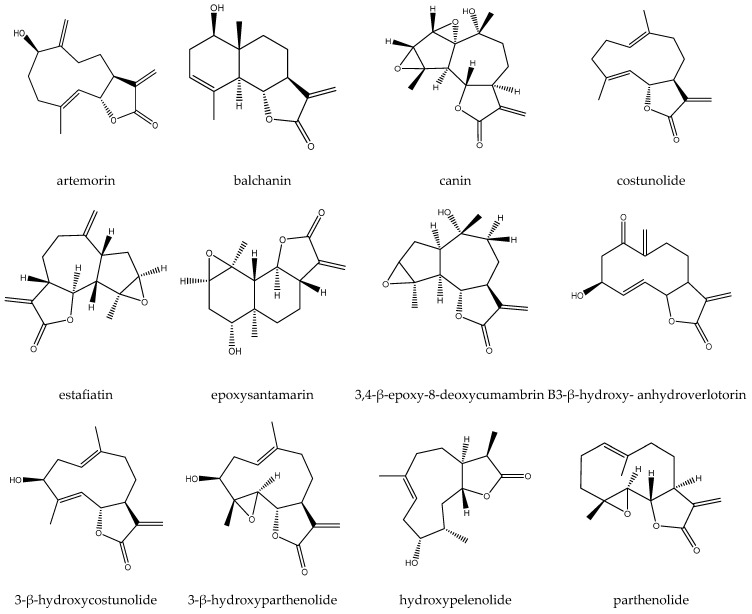

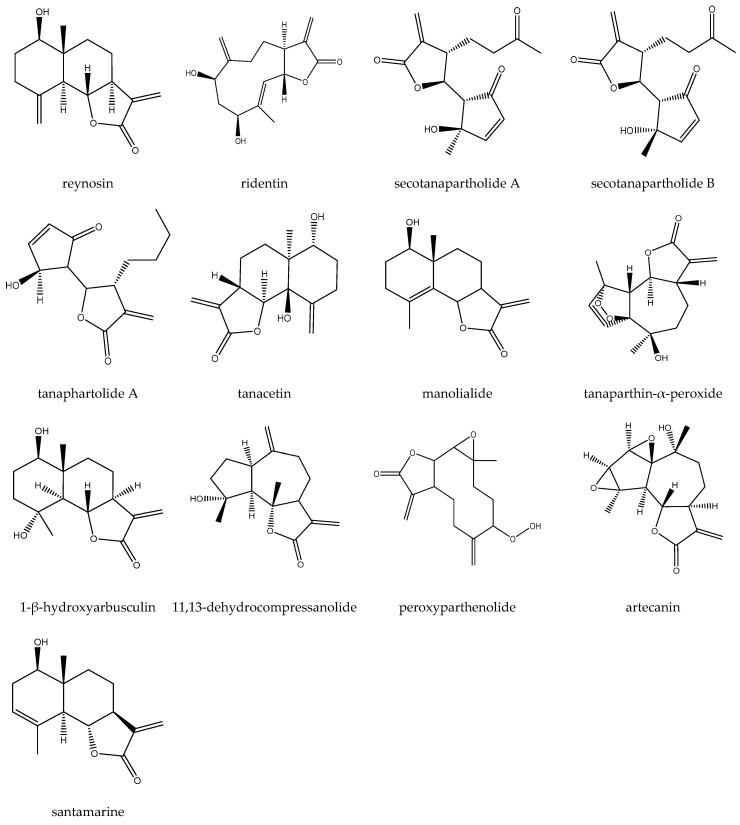
Sesquiterpene lactones isolated from feverfew.

Several sesquiterpene lactones (Figure 1) have been identified and reported, including artecanin, artemorin, balchanin, canin, costunolide, 11,13-dehydrocompressanolide, estafiatin, epoxysantamarin, 3,4-β-epoxy-8-deoxycumambrin B, 1-β-hydroxyarbusculin, 3-β-hydroxycostunolide, 3-β-hydroxyparthenolide, 3-β-hydroxy-anhydroverlotorin, hydroxypelenolide, parthenolide, peroxyparthenolide, reynosin, ridentin, secotanapartholide A, secotanapartholide B, santamarine, tanaphartolide A, tanacetin, manolialide, and tanaparthin-α-peroxide [6,10,11,12,13,14,15,16,17,18,19].

### 3.2. Flavonoids

Flavonoids are a class of polyphenolic secondary metabolites. Their general structure consists of a 15-carbon skeleton, with two phenyl rings and a heterocyclic ring abbreviated as C6-C3-C6.

**Figure 2:**
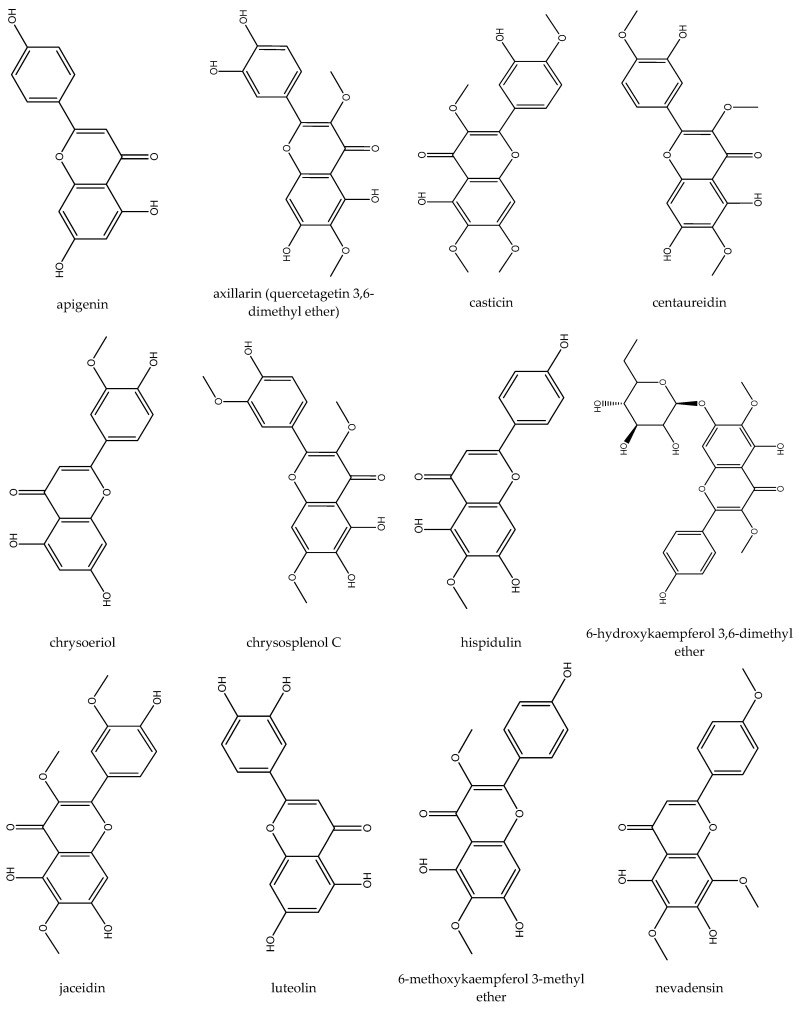

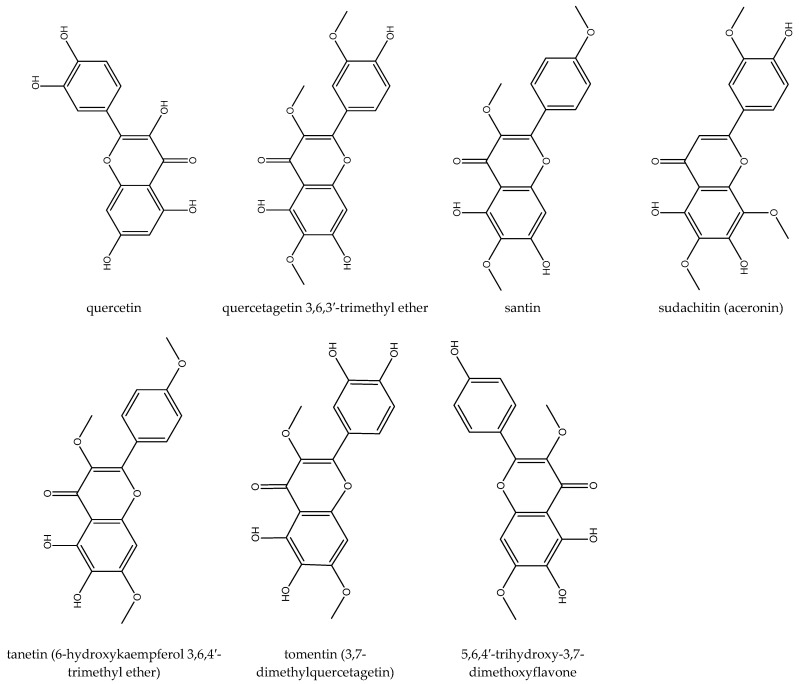
Flavonoids isolated from feverfew.

The following flavonoids have been isolated from feverfew: apigenin, axillarin, casticin, centaureidin, chrysoeriol, chrysosplenol C, hispidulin, 6-hydroxykaempferol 3,6-dimethyl ether, jaceidin, luteolin, 6-methoxykaempferol 3-methyl ether, nevadensin, quercetin, quercetagetin 3,6,3′-trimethyl ether, santin, sudachitin (aceronin), tanetin, tomentin, and 5,6,4′-trihydroxy-3,7-dimethoxyflavone (Figure 2). Also, some other flavonoids such as dihydroxy-dimethoxyflavone, trihydroxy-methoxyflavone, and trihydroxy-trimethoxyflavone have been reported [12,15,20,21,22,23,24,25,26,27].

### 3.3. Phytosterol and Triterpenoids

**Figure 3:**
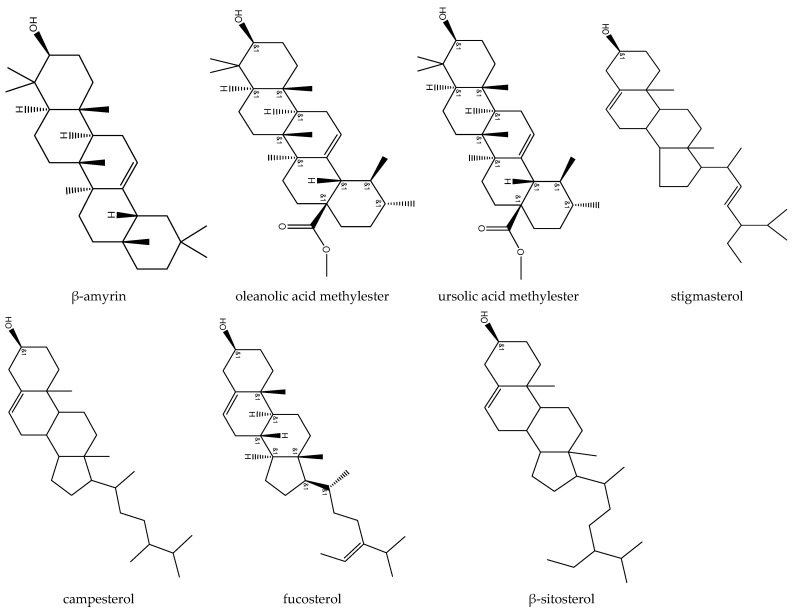
Phytosterol and triterpenoids isolated from feverfew.

Triterpenes are a class of secondary metabolites composed of three terpene units or six isoprene units. β-amyrin, oleanolic acid methyl ester, and ursolic acid methyl ester are triterpenoids reported from feverfew (Figure 3).

Phytosterols, inclusive of plant sterols and stanols, are steroid compounds found in plants that closely resemble cholesterol but differ in carbon side chains and the presence or absence of a double bond. Campesterol, fucosterol, β-sitosterol, and stigmasterol are phytosterols identified in feverfew extracts [12,28,29,30].

### 3.4. Essential Oil

**Figure 4:**
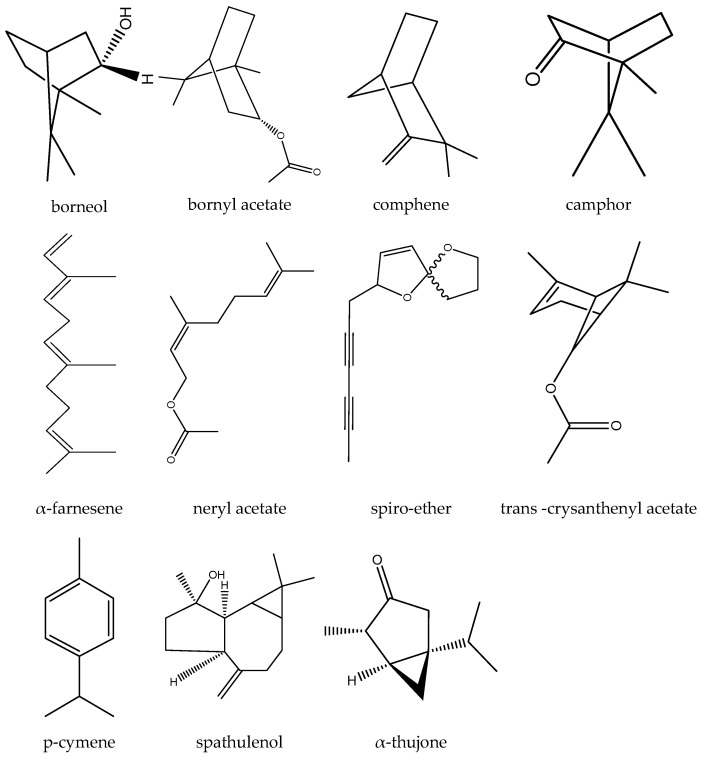
The major components of the essential oil obtained from feverfew.

Numerous studies in the literature have examined the essential oil content of both the roots and aerial parts of the plant through hydro-distillation or supercritical fluid extraction methods. The key findings reveal an array of compounds present in the essential oil of *T. parthenium* (Figure 4). For instance, camphor is a predominant component, varying in concentration from 27% to 75%, alongside other compounds like trans-crysanthenyl acetate, spiro-ether, neryl acetate, p-cymene, bornyl acetate, comphene, borneol, and α-thujone in the aerial parts [31,32,33,34,35,36]. On the other hand, the roots and rhizomes were found to contain camphor, (Z)-chrysanthenyl acetate, and α-farnesene as the major constituents [37].

Furthermore, a separate study highlighted the abundant presence of oxygenated monoterpenes (61.51%), in *T. parthenium* essential oil, with camphor and farnesol being the most prevalent compounds at 56.83% and 28.83%, respectively. The review of another survey identified the main volatile compounds in the essential oil as camphor, trans-chrysantenyl acetate, camphene, and cis-isogeraniol, comprising 45.47%, 21.65%, 9.48%, and 5.42% of the total oil composition, respectively. As evident from these studies, the chemical profiles of *T. parthenium* essential oil exhibit variations across different studies and regions due to factors such as harvesting locations, plant growth stages, environmental conditions such as climate and soil type, extraction methods, and drying techniques. Studies conducted in regions like Turkey, Kosovo, Tajikistan, and Iran have highlighted discrepancies in the predominant compounds like camphor, camphene, and chrisantenyl acetate, emphasizing the influence of both exogenous and endogenous factors on the volatile organic composition of the essential oil. Therefore, it is essential for future research endeavors to consider these variables when investigating the chemical composition of *T. parthenium* essential oil to enhance the understanding of its phytochemical diversity and geographical variations [37,38,39].

### 3.5. Fatty Acids

**Figure 5:**
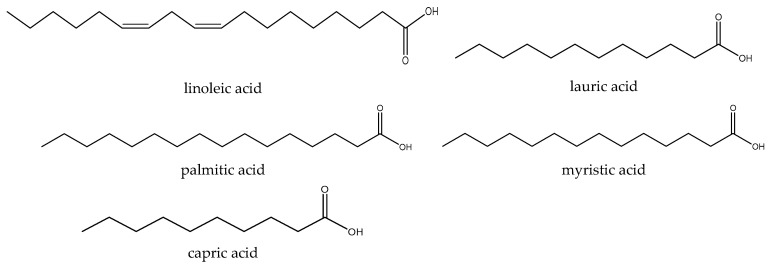
The major fatty acids identified in aerial parts of feverfew.

GC-MS has been used to investigate the lipophilic extract obtained from aerial parts of feverfew, and some fatty acids have been identified. Among them, palmitic acid, myristic acid, lauric acid, linoleic acid, and capric acid (Figure 5) were the primary fatty acids [21].

### 3.6. Phenolic Acids

**Figure 6:**
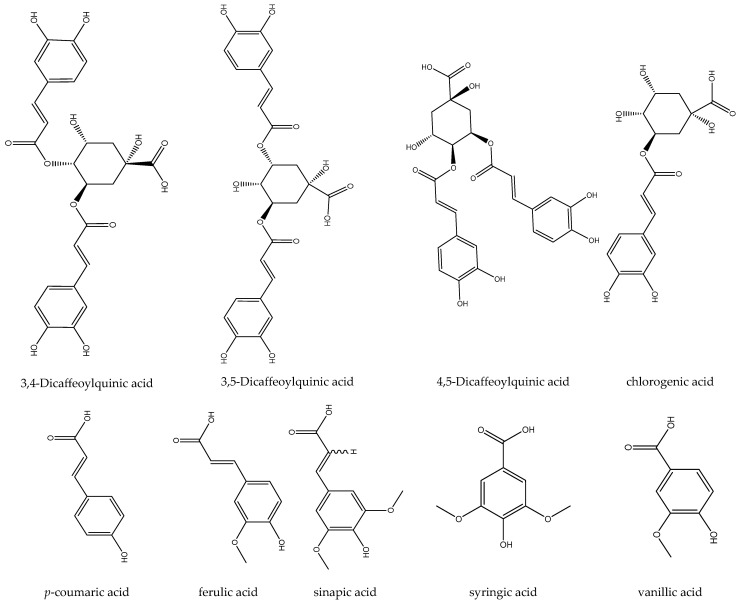
Some of the phenolic acids isolated from feverfew extract.

Some antioxidant phenolic acids, including 3,4-dicaffeoylquinic acid (isochlorogenic acid b), 3,5-dicaffeoylquinic acid, and 4,5-dicaffeoylquinic acid were identified in feverfew (Figure 6). di-O-caffeoylquinic acids (DCQAs) are polyphenolic acids that are classified into a large class of secondary plant metabolites known as phenylpropanoids [40]. Chlorogenic acid, syringic acid, ferulic acid, sinapic acid, vanillic acid, and *p*-coumaric acid are the other reported phenolic acids in feverfew [21].

### 3.7. Other Phytochemicals

Some other metabolites including the coumarin isofraxidin and 9-epipectachol B [41] have been isolated from the roots of the plant. Other reported metabolites are spiroketal enol ether diacetylenes [42,43], polyacetylenes, pyrethrin [44], (2-glyceryl)-O-coniferaldehyde [45], melatonin [46,47], and tannins [48].

## 4. Feverfew’s Anti-Inflammatory and Antioxidant Properties

Several studies have explored the mechanisms and signaling pathways related to feverfew’s anti-inflammatory and antioxidant properties. Table 1 provides an overview of the various mechanisms and signaling pathways associated with these properties. The key mechanisms related to anti-inflammatory effects are categorized into four groups as outlined below.

### 4.1. Inhibition of Pro-Inflammatory Enzyme Activity

5-Lipoxygenase is associated with inflammation by producing leukotrienes, which attract immune cells [49]. Cyclic adenosine monophosphate (cAMP) is an anti-inflammatory messenger, inhibiting the release of pro-inflammatory mediators. Phosphodiesterase enzymes hydrolyze cAMP, leading to inflammation [50]. Therefore, the inhibition of 5-lipoxygenase, phosphodiesterase-3, and phosphodiesterase-4 is associated with anti-inflammatory activities.

### 4.2. Modulation of Pro-Inflammatory Mediators

Prostaglandin E2 (PGE_2_) is produced from arachidonic acid by cyclooxygenase, leading to immune cell activation and inflammation. Agents with selective PGE_2_ inhibitory effects were therefore introduced to manage inflammation [51]. Tumor necrosis factor (TNF)-α is considered a pro-inflammatory cytokine, regulating immune and inflammatory responses [52]. Consequently, TNF-α inhibitors, such as etanercept, infliximab, adalimumab, certolizumab pegol, and golimumab, have been in development to address inflammatory disease [53].

Interleukins (ILs) are cytokines with important roles in regulating the immune response. IL-1 is released from various cells and plays an important role in activating leukocytes, stimulating macrophage, and increasing the adhesion of leukocytes to the endothelial. IL-2 is involved in the inflammatory response, enhancing cytokine synthesis. IL-12 is produced by monocytes, inducing interferon-gamma production. On the other hand, IL-10 inhibits the production of interferon-gamma and IL-2, serving as an anti-inflammatory cytokine [54].

### 4.3. Modulation of Adhesion Molecule Expression

Intercellular adhesion molecule-1 (ICAM-1) is associated with leukocyte migration by mediating leukocyte adhesion to endothelial cells [55]. Therefore, a reduction in the expression of ICAM-1 is considered in developing anti-inflammatory agents.

### 4.4. Inhibition of NF- kB

The NF-κB signaling pathway is critical in regulating inflammation. The activated NF-κB upregulates the expression of pro-inflammatory cytokines, such as interleukin-1, and adhesion molecules, which results in inflammation [56].

**Table 1:** Key mechanisms and signaling pathways related to feverfew’s anti-inflammatory and antioxidant properties.

Mechanism of Action		Extract/Component	Study Type	Outcomes	Ref.
Anti-inflammatory effect	Modulation of adhesion molecule expression	Parthenolide	in vitro/human RA synovial tissue	↓Intercellular adhesion molecule-1 (ICAM-1)	[57]
	Inhibition of pro- inflammatory enzyme activity	Parthenolide	in vivo In vitro/human epidermal keratinocytes	↓5-lipoxygenase (IC50:11.8 ± 4.8 µg), phosphodiesterase-3 (IC50: 35.2 ± 12.3 µg/mL), and phosphodiesterase-4 (IC50: 20.8 ± 9.4 µg/mL)	[58]
	Modulation of pro- inflammatory mediators	Parthenolide	in vivo in vitro/human epidermal keratinocytes	↓PGE2 production (IC50: 37.9 ± 4.16 µg/mL) and TNFa production (IC50: 31 ± 0.04 µg/mL)	[58]
		Parthenolide	in vitro/RAW264.7 cells	↓IL-12 production	[59]
		Aqueous extract	in silico	↓ PGE2 and IL-1β, ↑ IL-10	[60]
		Parthenolide	in vitro/human RA synovial tissue	↓TNF-α, and IFN-gamma	[57]
		Feverfew’s extracts	in vitro/human monocytic THP-1 cells	↓ LPS-mediated TNF-α and CCL2 (MCP-1)	[61]
	Inhibition of NF- kB	Parthenolide	in vitro/peripheral blood T cells	Inhibition of NF- kB	[62]
Antioxidant activity		Parthenolide	in vivo	↑Total antioxidant capacity, glutathione (GSH) content, superoxide dismutase (SOD), and catalase (CAT) ↑The survival rate in mice pretreated with parthenolide compared to the control group when exposed to electron beam irradiation	[63]
		Methanol extract	in vitro	↑Confirmed by evaluation by 1,1-diphenyl-2-picryl-hydrazyl (DPPH) assay	[64]

## 5. Feverfew’s Medicinal and Therapeutic Properties

### 5.1. Neurological Disorders

#### 5.1.1. Migraine

Several clinical trials have been conducted on the migraine prophylactic effect of feverfew and its bioactive components, especially parthenolide [65,66,67,68,69,70]. A clinical study showed that migraine sufferers would benefit from feverfew, especially formulations containing a controlled amount of sesquiterpene lactone [65]. Based on a randomized double-blind placebo-controlled clinical trial, the frequency and severity of migraine attacks were reduced in patients who took a capsule of dried feverfew leaves (2.19 µmol parthenolide/dose) once a day [66]. In another double-blind, randomized, placebo-controlled clinical study, patients who consumed capsules containing 6.25 mg of MIG-99, a CO(2) extract of feverfew, experienced the highest level of benefit compared to those in the groups taking capsules with 2.08 mg and 18.75 mg [68]. A clinical trial also demonstrated that the frequency of migraine attacks decreased from 4.76 to 1.9 attacks per month in patients taking 6.25 mg of MIG-99 three times a day [69]. Moreover, the headache intensity and pain frequency were reduced in children who suffered from migraine and tension-type headaches after taking a nutraceutical formulation containing feverfew, *Andrographis paniculata*, CoQ10, riboflavin, and magnesium [70].

In support of the therapeutic effects of this herb, various in silico, in vitro, and in vivo studies have been conducted, leading to an increase in our knowledge of underlying signal pathways and mechanisms.

As microglia activation is considered the reason for migraine attacks, potential anti-migraine medicines would be able to inhibit glial activation and block neuroinflammatory release. An in vitro study showed that the release of pro-inflammatory cytokine, including IL-6 and TNF-α, and the translocation of NF-κB to the nucleus cell was reduced in lipopolysaccharide (LPS)-stimulated BV-2 microglia pretreatment with parthenolide [71]. An in vivo study showed that parthenolide inhibited NF-κB and nitroglycerin-induced Fos expression in the nucleus trigeminalis caudalis and brain nuclei [72].

Nitric oxide (NO), a signaling molecule, plays an important role in biological activities. Based on numerous pieces of evidence, the components of the NO signaling cascade are upregulated in migraine sufferers [73]. An in vitro study demonstrated that parthenolide can inhibit NO/iNOS synthesis and inhibit the activation of p42/44 MAPK in rat microglia cells [74]. Following this, a study proved that the supercritical extract of feverfew would inhibit the production of NO due to the downregulation of both eNOS and iNOS enzymes. This study also showed that the TNF-α production was reduced in a dose-dependent manner [75].

Neuropeptide calcitonin gene-related peptide (CGRP) released from trigeminal neurons is described as one of the most important migraine triggers [76]. Stimulating transient receptor potential (TRP) channels, such as ankyrin 1 (TRPA1), induces CGRP release and migraine attacks. As a result, TRPA1 antagonisms can be anti-migraine agents [77]. A study demonstrated that parthenolide is a partial TRPA1 agonist and desensitizes this channel after initial stimulation [78]. Additionally, serotonin antagonists can act as anti-migraine agents since binding serotonin to its 5-HT3 receptor mediates CGRP release [79]. A study showed that feverfew powder containing an acceptable amount of parthenolide possesses anti-serotonergic properties [80].

#### 5.1.2. Epilepsy

Feverfew with honey is traditionally prescribed as an anti-epileptic drug in Danish folk medicine, which possibly acts through the GABAergic system. An in vitro study showed that the ethanol extract of feverfew has a good affinity to the benzodiazepine site of GABA receptors [81]. Another study proved that feverfew extracts containing apigenin had the highest affinity to the GABA_A_-benzodiazepine site [82].

#### 5.1.3. Neuroprotective Effects

Several studies have been conducted on the neuroprotective activity of feverfew. Based on a study, parthenolide activates the protein kinase B (Akt)/glycogen synthase kinase-3β (GSK-3β) signaling pathway, reduces the expression of HIF-1α expression, and inhibits apoptosis in oxygen–glucose deprivation (OGD)-induced apoptosis PC12 cells [83]. A study on pentylenetetrazol (PTZ)-induced seizures in mice showed that a medium dose of feverfew extract attenuates brain oxidative damage through decreasing malondialdehyde (MDA) levels and rising superoxide dismutase (SOD) and catalase (CAT) activity [84]. The neuroprotective effect of parthenolide was evaluated in mice suffering from traumatic brain injury. This study demonstrated that parthenolide can prevent microglial activation and reduce pro-inflammatory cytokines production. Additionally, it was shown to inhibit the activation of STAT3/NF-κB and the inflammasome [85]. Another study revealed that parthenolide could be effective in addressing neuroinflammation and safeguarding cells against ischemic brain injury through the regulation of the RhoA/ROCK pathway, ultimately resulting in the control of microglial polarization [86].

#### 5.1.4. Parkinson’s Disease

An animal study revealed that parthenolide, ACT001, with low doses of L-DOPA reduced dopaminergic neurodegeneration and decreased the overexpression of α-synuclein, activation of astrocyte, and production of IL-1β in the substantia nigra and striatum of a mice brain suffering from 1-methyl-4-phenyl-1, 2, 3, 6-tetrahydropyridine (MPTP)-induced Parkinson’s disease. This study also showed that ACT001 is an anti-apoptotic agent through activating the Caspase3, elevating the amount of the anti-apoptotic signaling Bcl-2 molecule and the pAkt/Akt ratio as well as deactivating pro-apoptotic signaling molecule Bax in the substantia nigra and striatum [87]. Another study demonstrated that the feverfew extract inhibits monoamine oxidase B (MAO-B) (0.4 > IC_50_ < 0.7 mg/mL) [88].

#### 5.1.5. Neuropathic Pain

In a study evaluating the impact of *T. parthenium* on neuropathic pain, a randomized controlled trial was carried out involving 48 adult male Wistar rats. Chronic constriction injury was induced on the left sciatic nerve to simulate neuropathic pain in the rats. The administration of *T. parthenium* extracts via intragastric tube on a daily basis for a duration of 2 weeks resulted in a significant improvement in pain symptoms. The assessment of mechanical allodynia and thermal hyperalgesia using Von Frey hairs and a plantar test device showed promising outcomes. The *T. parthenium* extract demonstrated a remarkable increase in the paw withdrawal threshold in response to mechanical stimulation, indicating a reduction in pain sensitivity. Additionally, there was a noticeable improvement in paw withdrawal latency in response to thermal stimulation, further supporting the analgesic effect of *T. parthenium* extract [89].

In another animal study involving streptozotocin (STZ)-diabetic rats, the antihyperalgesic properties of feverfew flower extract were investigated, demonstrating its potential in mitigating neuropathic pain. Notably, the active compound identified as parthenolide, a prominent constituent of the feverfew plant, played a pivotal role in exerting this effect. The administration of the feverfew flower extract at a dose of 1000 mg/kg resulted in a partial reversal of mechanical hyperalgesia in STZ-treated rats, with a time-dependent antihyperalgesic response observed. Conversely, the feverfew leaf extract did not exhibit efficacy in alleviating neuropathic pain, emphasizing the necessity of higher concentrations of parthenolide for nociception improvement in diabetic rats. The findings emphasize feverfew extracts’ potential for managing painful diabetic peripheral neuropathy, promising further clinical investigation and development [90].

#### 5.1.6. Anxiety and Depression

Based on an in vivo study conducted by Cárdenas et al., the aqueous extract of *T. parthenium* demonstrated significant anxiolytic and antidepressant effects [91]. Anxiolytic effects were observed in the Burying Behavior Test (BBT), where *T. parthenium* at doses of 5, 10, and 20 mg/kg reduced burying behavior and increased latency, akin to the results produced by Diazepam at 0.5 mg/kg. In the Elevated Plus Maze Test (PMT), *T. parthenium* showed anxiolytic effects by increasing the time spent in open arms at doses of 0.5, 1, 5, and 10 mg/kg, similar to standard drugs such as Diazepam and Alprazolam. Additionally, in the Forced Swimming Test (FST), *T. parthenium* doses of 10, 20, and 40 mg/kg significantly reduced immobility time, indicating antidepressant-like effects comparable to those of Alprazolam. These findings suggest that *T. parthenium* has potential therapeutic benefits in anxiety and depression, possibly mediated through the GABAergic system. The study supports the traditional use of *T. parthenium* and highlights its promise in addressing anxiety and depression disorders, providing valuable insights for its further exploration in therapeutic applications [91].

#### 5.1.7. Hypnotic

An animal study showed that feverfew holds hypnotic properties by decreasing the latency of sleep and prolonging sleep time due to its flavonoid content [92].

### 5.2. Cancer

There is a great deal of evidence on the anticancer pharmacological properties of feverfew and its major bioactive component (parthenolide) [93].

Based on an in vitro study, parthenolide possesses antiproliferation properties on human lung carcinoma (A549), human medulloblastoma (TE671), human colon adenocarcinoma (HT-29), and human umbilical vein endothelial cells (HUVECs), confirmed by a 3-(4,5-dimethylthiazol-2-yl)-2,5-diphenyltetrazolium bromide (MTT) test [94]. Mutation in the B-raf proto-oncogene (*BRAF*) gene that encodes B-raf proteins is associated with the development of various cancers. This protein is an important part of the MAPK/ERK signaling pathway [95]. Signal transducer and activator of transcription (STAT) 3 is recognized as an important transcription regulator, playing a multifaceted role in developing cancer. As a result, STAT3 inhibitors may show anticancer activity in the specific type of tumors [96]. According to another in vitro study, parthenolide is a strong cytotoxic and antiproliferative agent against non-small cell lung cancer cells by reducing the B-Raf expression in both protein and mRNA levels. This study also showed that the activity of STAT3 is inhibited by parthenolide [97].

Matrix metalloproteinases (MMPs) are proteases that play an important role in the metastasis, proliferation, and growth of cancerous cells. The elevated levels of MMPs mediate the degradation of the extracellular matrix and blood vessels, leading to cancer metastasis. For example, trans-membrane-type 4 MMP (MT4-MMP) increases cell migration and invasion, and MT1-MMP induces tumor growth through the activation of pro-MMP-2. Transforming growth factor-β (TGF-β) is among the most important epithelial-to-mesenchymal transition (EMT) activators, resulting in metastasis through the upregulation of MT1-MMP and downregulation of E-cadherin (E-cad). N-cad is induced by the E-cad downregulation, which leads to cell proliferation through MAPK pathway activation [98]. In a study on colorectal cancer cells (SW620), parthenolide suppressed the MAPK signaling pathway and downregulated EMT markers (vimentin, β-catenin, and Snai), MMPs (MMP-2 and MMP-9), and COX-2 expressions. However, the expression of E-cad was upregulated [99]. Additionally, TWIST1 expression would lead to EMT, which is directly influenced by TGF-β1 [100]. Based on in silico and in vitro studies, parthenolide downregulates the expression of TGF-β1 and TWIST1 in breast cancer cells [101].

Focal Adhesion Kinase 1 (FAK1) is a non-receptor tyrosine kinase dysregulated in various aspects of cancer development and progression. The overexpression of FAK1 is related to the epithelial to mesenchymal transition (EMT) and tumor angiogenesis [102]. A study showed that parthenolide inhibited the FAK1 signaling pathway in breast cancer cells [103].

NF-κB plays an important role in the pathogenesis and treatment of cancers. In normal conditions, NF-κB is inactivated by forming NFκB–IκBα or NFκB–IκBε complexes. After induction by some stimuli, the IκB kinase (IKK) complex triggers the degradation of IκB and activation of NF-κB [104]. The activated NF-κB, is associated with the regulation of anti-apoptotic proteins, such as Bax and Bad, TNF-receptor-associated factors (TRAFs), and pro-apoptotic proteins, such as Bcl-2. Based on an in vitro study, parthenolide inhibits NF-κB activity in a dose-dependent manner in multidrug-resistant MDA-MB-231-BCRP cells [105]. There is also evidence that parthenolide can increase the sensitivity of gastric cancer cells to chemotherapy through the inhibition of the NF-κB pathway as well as the induction of apoptosis [106]. An in vitro study revealed that parthenolide can inhibit angiogenesis [107]. NF-κB is also correlated with angiogenesis through the activation of an angiogenesis-promoting factor, which is named VEGF. An in vivo study showed that the parthenolide anti-angiogenic effect is mediated through the inhibition of the NF-κB signaling pathway [108].

Additionally, the insulin-like growth factor 1 receptor (IGF-1R) is recognized as the kinase target in cancer treatment [109]. IGF-1R is overexpressed in tumor cells and mediates several signal pathways, such as phosphatidylinositol 3-kinase (P13K) /protein kinase B (AKT), leading to proliferation and metastasis [110]. The activated AKT is involved in transformation by phosphorylating FoxO3α [111]. Both in vivo and in vitro studies have shown that parthenolide can reduce lung cancer cell growth by inhibiting IGF-1R/PI3K/Akt/FoxO3α signaling pathway [112].

Microtubule-interfering agents, such as paclitaxel, vincristine, and vinblastine are among the most effective anticancer medicines. Microtubule disruption results in various cellular responses, leading to cell cycle arrest or cell death [113]. In an in vitro assay, parthenolide showed antimicrotubular and antiproliferative properties in human breast cancer MCF-7 cells [114].

### 5.3. Respiratory System Disorders

#### 5.3.1. Asthma

In a murine model of chronic asthma, a study conducted by Arıkan-Ayyıldız et al. aimed to explore the effects of parthenolide, the primary compound found in *T. parthenium* [115]. The research involved thirty-five BALB/c mice assigned to five groups: a control group (I), a placebo group (II), a group receiving dexamethasone (III), a group receiving parthenolide (IV), and a group receiving a combination of dexamethasone and parthenolide (V). Mice in group III were given dexamethasone at 1 mg/kg, while those in group IV received parthenolide at 3 μg/g. Group V received both dexamethasone (1 mg/kg) and parthenolide (3 μg/g), and group II was given 50 μL of DMSO, the solvent for parthenolide. All treatments were administered once daily for the last seven days of the challenge period. The study found that parthenolide improved histologic parameters, except for the mast and goblet cell counts, compared to placebo. However, dexamethasone showed better results in most histological parameters than parthenolide alone. Combining parthenolide and dexamethasone did not offer additional benefits or synergistic effects over dexamethasone treatment alone. Parthenolide also reduced IL-4 levels significantly compared to placebo, suggesting its potential therapeutic impact in ameliorating certain pathological alterations in asthma. However, as a standalone intervention, parthenolide was less effective than dexamethasone [115].

#### 5.3.2. Acute Lung Injury

Another study revealed that parthenolide could alleviate inflammation and decrease pulmonary macrophages (M phenotype) by inhibiting NF-κB and STAT1 signaling pathways in an animal acute lung injury model [116].

#### 5.3.3. Acute Respiratory Distress Syndrome

The therapeutic potential of parthenolide in acute respiratory distress syndrome was confirmed in an animal model study, suggesting its effect on regulating cytokine release and improving autophagy [117].

### 5.4. Skin Disorders

There is a general belief that feverfew could protect skin against various environmental factors, such as ultraviolet irradiation. The skin protection of parthenolide-depleted extract of feverfew against UV was confirmed by both in vitro and in vivo tests. This study also showed that this extract holds radical scavenging activity against free radicals [118]. An in vitro study suggested that feverfew can be a part of cosmetic products. This study showed that feverfew’s sesquiterpene lactones inhibit melanin biosynthesis through the downregulation of tyrosinase expression in mouse B16 melanoma cells [119].

### 5.5. Anti-Fibrotic Effects

A study showed that parthenolide has the potential to address the core of peritoneal fibrosis by inhibiting the TGF-β/Smad pathway. This study also confirmed that the expression of fibrotic markers (fibronectin and collagen I) was suppressed by parthenolide in peritoneal dialysis-related peritoneal fibrosis [120]. Another in vitro study confirmed the anti-fibrotic properties of parthenolide through NF-κB signaling pathway suppression in primary fibroblasts derived from patients either suffering from idiopathic pulmonary fibrosis or not [121]. Parthenolide alleviated pulmonary fibrosis induced by bleomycin in rats, mostly attributed to its inhibitory effects on the NF-κB/Snail signaling pathway [122].

### 5.6. Ulcerative Colitis

A survey indicated that parthenolide can suppress the NF-κB signaling pathway and pro-inflammatory cytokine release, leading to its therapeutic effect in managing ulcerative colitis [123].

### 5.7. Anti-Osteoclastogenic Effects

The therapeutic effect of parthenolide in managing periodontitis was confirmed in human periodontal ligament-derived cells. This study showed that parthenolide inhibited the activation of NF-κB and ERK while also reducing the expression of osteoclastogenic and inflammatory genes, such as IL-1β, IL-6, TNF-α, RANKL, OPG, and M-CSF [124].

### 5.8. Endometriosis

A study demonstrated that parthenolide decreased vimentin (mesenchymal marker) levels, increased E-cadherin (epithelial marker) levels and suppressed the PI3K/AKT/GSK-3β/nβ-catenin signaling pathway in rats with endometriosis [125].

### 5.9. Obesity

Research on parthenolide from *T. parthenium* has revealed its anti-obesity properties by regulating inflammatory responses. In vitro studies showed that parthenolide reduced IL-6 and MCP-1, modulated adiponectin and resistin levels, and activated Nrf2/HO-1 signaling pathway. In high-fat diet mouse models, its administration led to reduced body weight and white adipose tissues through NF-κB and MAPK regulation. Parthenolide also influenced pro-/anti-inflammatory markers in macrophages and boosted antioxidant defenses via Nrf2/Keap1 signaling. Overall, parthenolide showed promise in combating obesity-related ailments by targeting inflammation and oxidative stress pathways [126].

### 5.10. Antiprotozoal Effect

An in vitro study showed that the hydroalcoholic extract of feverfew possesses antiprotozoal activity against amastigote and promastigote forms of *Leishmania amazonensis* and epimastigote form of *Trypanosoma cruzi* [127]. Another in vitro study also confirmed the leishmanicidal activity of parthenolide [128]. The antiprotozoal activity of parthenolide against epimastigote forms of *Trypanosoma cruzi* was evaluated and confirmed in an in vitro study [129]. Guaianolide that was purified from the hydroalcoholic of feverfew also showed antiprotozoal activity against *Trypanosoma cruzi* [130].

## 6. Safety, Toxicity, and Adverse Effects of Feverfew

In the case of the safety of the administration of feverfew-containing products in pregnancy/lactation, avoiding their use is suggested. The plant should not be used during pregnancy since the leaves have emmenagogic properties, which might lead to the expulsion of the placenta and embryonic membranes, perhaps resulting in abortion. Moreover, it is advised to avoid feverfew during breastfeeding or in children under 2 years of age [131,132,133].

Drug interactions with feverfew are not well documented, but there is a potential for interactions when it is coadministered with substances such as anticoagulants, antiplatelet agents, nonsteroidal anti-inflammatory drugs (NSAIDs), salicylates, or thrombolytic agents [131,134,135,136]. 

Feverfew should not be used in individuals who have allergies to other plants in the Asteraceae family, including aster, chamomile, chrysanthemum, ragweed, sunflower, tansy, and yarrow [137,138,139]. Given its potential antiplatelet effects [134,135], it is advised against using it in patients who are undergoing surgery. Individuals suffering from coagulation disorders should obtain advice from their healthcare professional before using any products that contain feverfew [131].

Patients who discontinue the use of feverfew may encounter a phenomenon sometimes referred to as ‘post-feverfew syndrome’ [140]. Direct contact with fresh feverfew leaves may result in allergic contact dermatitis. Reports have indicated that feverfew consumption in some individuals might lead to swelling of the lips, tongue, and oral mucosa, as well as mouth ulcers. Gastrointestinal symptoms, such as abdominal discomfort, nausea, vomiting, diarrhea, indigestion, and flatulence, may also manifest [139,141].

Parthenolide is a strong contact allergen [139,142,143]. In the case of allergic diagnostic tests, patch testing either with parthenium extract or with sesquiterpene lactones (SQLs) is the standard diagnostic test in clinically suspected cases of parthenium dermatitis. Both parthenolide (0.1% pet.) and SQL mix (0.1% pet.), a mixture of alantolactone, dehydrocostus lactone, and costunolide, are included for patch testing in most plant series. A study was performed to evaluate whether parthenolide detects parthenium contact sensitivity more effectively than parthenium extract in patients clinically suffering from classic parthenium dermatitis. It was concluded that patch testing with parthenolide (0.5% pet.) detects fewer cases of suspected parthenium dermatitis than patch testing with parthenium extract (1% aq.) [137,141].

Parthenolide is an alkylating sesquiterpene lactone due to the presence of an α-methylene-γ-butyrolactone moiety and an electrophilic epoxide group in its structure. These elements are reactive towards thiols in proteins, which could elucidate its anti-inflammatory and cytotoxic properties, along with its allergenic potential. In a phase I dose escalation trial, as an anticancer candidate, parthenolide at a daily dose of up to 4 mg orally was well tolerated in patients. There was no detectable concentration in the plasma, with a detection limit of 0.5 ng/mL [142]. Since parthenolide has been introduced as a potent skin sensitizer, to eliminate the risk of skin sensitization from feverfew, some studies have investigated a parthenolide-depleted extract of feverfew (PD-Feverfew) or parthenolide-free extract (PFE-Feverfew) and reported their effectiveness as an anti-inflammatory agent [144]. It was also suggested that the parthenolide-depleted extract of feverfew may protect skin from UV irradiation and external aggression [118].

There have been no investigations conducted on the long-term harmful effects of feverfew; thus, the long-term safety of feverfew-containing products has not been determined. But overall, the data suggest that feverfew is associated with only mild and transient adverse effects [145].

## 7. Guidelines for Healthcare Providers on the Use of Feverfew

This section provides essential guidance and recommendations for healthcare providers and patients on the appropriate use of feverfew, based on the information from the preceding section on the safety, toxicity, and adverse effects of feverfew:

✓ Pregnancy and Lactation: Avoid feverfew during pregnancy and breastfeeding due to potential risks.
✓ Pediatric Use: Not recommended for children under 2 years of age.
✓ Drug Interactions: Caution advised with anticoagulant medications and NSAIDs.
✓ Allergies: Be wary if allergic to plants in the Asteraceae family.
✓ Pre-surgery: Discontinue use before surgery to prevent bleeding complications.
✓ Monitoring Adverse Effects: Report any unusual symptoms promptly.
✓ Long-term Use: Lack of data on prolonged safety—regular monitoring recommended.
✓ Parthenolide-free Alternatives: Consider using parthenolide-free extracts for reduced allergic risk.

## 8. Conclusions

Feverfew has been widely prescribed since ancient times. This herb contains various metabolites extracted from different parts of this medicinal plant. The primary phytochemical classes of feverfew are sesquiterpene lactones and flavonoids. Additionally, monoterpenes, phytosterols, and fatty acids contribute to the plant’s bioactive components. Parthenolide, the most bioactive compound, is crucial in leading to a wide range of pharmacological effects.

Feverfew has been evaluated for numerous therapeutic applications, notably for managing neurological disorders and cancers. Moreover, it has a positive impact on the management of inflammatory diseases, such as asthma, ulcerative colitis, and endometriosis.

Among the various signaling pathways implicated in the pharmacological and therapeutic activities of feverfew, NF-κB stands out prominently. Additionally, the MAPK and Akt signaling pathways are associated with the effects of feverfew. Therefore, drug design studies are recommended to develop targeted medicines containing feverfew, aiming to efficiently address various medical conditions.

Future studies should delve into the synergistic effects of *T. parthenium*’s bioactive components on specific signaling pathways. More exploration of additional signaling pathways beyond NF-κB, MAPK, and Akt could unveil novel molecular targets for drug development. Understanding the molecular mechanisms of feverfew’s actions can drive the design of personalized and potent therapeutic interventions across various medical conditions.

## Data Availability

Not applicable.
